# Prognostic Value of Nicotinamide N-Methyltransferase Expression in Patients With Solid Tumors: A Systematic Review and Meta-Analysis

**DOI:** 10.3389/fphys.2018.01407

**Published:** 2018-10-08

**Authors:** Shimeng Li, Lu Qiao, Zhaowei Yang, Chengyan He

**Affiliations:** Department of Clinical Laboratory, China-Japan Union Hospital of Jilin University, Changchun, China

**Keywords:** meta-analysis, solid tumor, nicotinamide N-methyltransferase (NNMT), prognosis biomarker, poor outcome

## Abstract

**Background:** Nicotinamide N-methyltransferase (NNMT) is an enzyme that catalyzes N-methylation of pyridine-containing compounds. NNMT is upregulated in many types of solid tumors, suggesting the potential for its use as a tumor biomarker. However, the prognostic value of NNMT in solid tumors is still unclear. We therefore conducted a meta-analysis to investigate the association between NNMT expression and survival in patients with solid tumors.

**Methods:** We focused on patients with solid tumors, using high NNMT expression levels as the intervention and low NNMT expression levels as the comparison, according to Patient, Intervention, Comparison, and Outcome (PICO) guidelines. Electronic databases (up to June 7, 2018) were comprehensively searched to collect relevant cohort studies regarding the associations between NNMT expression levels and survival outcomes (overall survival [OS], disease-specific survival [DSS] including cancer-specific survival [CSS], and time to tumor progression [TTP] including disease-free survival [DFS], progression-free survival [PFS], and metastasis-free survival [MeFS]). Publication biases were also examined. All analyses were performed using STATA 12.0 software.

**Results:** A total of 3340 patients with solid tumors from nine published studies were included. The combined hazard ratio (HR) identified high NNMT expression levels as a poor prognostic predictor of OS (HR = 1.67, 95% CI = 1.23–2.26). However, NNMT levels had no significant association with DSS (HR = 1.47, 95% CI = 0.95–2.28) and TTP (HR = 1.13, 95%CI = 0.39–3.25).

**Conclusion:** High NNMT expression levels may be a poor prognostic biomarker for patients with solid tumors.

## Introduction

Cancer is one of the leading causes of morbidity and mortality worldwide. In 2012, approximately 8.2 million deaths occurred due to cancer (Torre et al., 2015). Global Health Observatory (GHO) data showed that cancer resulted in 8.8 million deaths in 2015. Genomic and epigenomic changes have been implicated as a critical factor in the oncogenic transformation of normal cells. Epigenetic modifications, including the methylation and acetylation of histones, as well as DNA methylation, have been shown to be involved in the development and progression of many types of cancer (Sui et al., 2015). Abnormal alterations in histone acetylation are associated with cancer development (for example, loss of acetylation at lysine 16) (Li and Seto, 2016). Abnormal methylation in promoter regions of genes have been found in cancers, dysregulating expressions of such genes (Herman and Baylin, 2003). Nicotinamide N-methyltransferase (NNMT) is essential for histone methylation, and its expression results in dysregulated transcription and translation of several key genes involved in the development of glioblastoma. NNMT is involved in reorganizing the methylome in glioblastoma cells, as NNMT inhibition upregulates the availability of methyl groups that leucine carboxyl methyl transferase 1 uses to methylate protein phosphatase 2A, further inhibiting serine/threonine kinases (Palanichamy et al., 2017). Elevated mRNA levels of NNMT have been identified in six major databases, including TCGA GBM, Rembrandt, TCGA GBMLGG, Phillips, Gravendeel, and Nutt. NNMT protein levels are higher in tissues taken from glioblastoma patients compared with those from patients with nonmalignant brain tumors (Jung et al., 2017). Moreover, NNMT dysregulation has been shown to be a contributing factor to the progression of gastric cancer, pancreatic cancer, prostate cancer, nasopharyngeal cancer, lung cancer, hepatocellular cancer, and renal cancer (Yao et al., 2005; Kim et al., 2009; Ujiie et al., 2012; Win et al., 2013; Bi et al., 2014; Zhou et al., 2014; Chen et al., 2016; Xu et al., 2016). Interestingly, high NNMT expression levels correlate with poor prognosis in patients with glioblastoma (Jung et al., 2017), gastric cancer (Chen et al., 2016), and pancreatic cancer (Xu et al., 2016). However, results obtained from these studies did not show a statistically significant correlation between NNMT levels and prognosis in patients with glioblastoma (Jung et al., 2017), pancreatic cancer (Bi et al., 2014), and lung cancer (Ujiie et al., 2012). Thus, the prognostic value of NNMT in solid tumors remains controversial.

To determine the prognostic value of NNMT expression levels in patients with solid tumors, we selected nine articles from 789 references and investigated the association between NNMT expression levels and the prognosis of patients with solid tumors.

## Materials and methods

This study was performed according to the Preferred Reporting Items for Systematic Reviews and Meta-Analyses (PRISMA) guidelines. It was registered in PROSPERO, and the registration number is CRD42018088951.

### Search strategy and selection criteria

Electronic databases, including PubMed, Embase, and Web of Science were queried for studies (written in English) regarding NNMT expression levels and the survival of patients with solid tumors. The China National Knowledge Infrastructure, the Wanfang Data, and the Chinese Scientific Journals Database were searched for publications in Chinese. All databases were updated to June 7, 2018. NNMT or nicotinamide N-methyltransferase was used as search term. Other relevant studies were also retrieved from references of selected articles.

In accordance with Patient, Intervention, Comparison, Outcome and Study Design (PICOS) guidelines, we focused on patients with solid tumors. High NNMT expression levels and low NNMT expression levels were regarded as intervention and comparison, respectively. Clinical outcomes included overall survival (OS), disease-specific survival (DSS), and time to tumor progression (TTP). Studies involved in cohort study design were included. We collected all eligible articles evaluating the association between NNMT expression levels and clinic outcome. All included studies had to meet the following criteria: (1) they evaluated the prognosis of patients with solid tumors; (2) they provided a hazard ratio (HR) and 95% confidence interval (CI), or included sufficient information to estimate these parameters; and (3) a cut-off value was given to stratify the expression levels as “high” and “low.” Exclusion criteria were as follows: (1) letters, reviews, experimental studies, case reports, conference abstracts, and duplicated studies; (2) NNMT expression levels detected in cell lines; and (3) studies that did not provide enough data to calculate the HR and 95% CI.

### Data extraction

Two investigators (SL and LQ) independently extracted relevant information from the eligible studies according to predefined data in abstraction form. Discrepancies were resolved through discussions between SL and LQ. Where there was a disagreement, the issue was resolved by discussion or consensus with the third investigator (ZY). We collected the following details: name of the first author, publication year, region, type of cancer, number of cases (high expression/low expression), follow-up period, test method, location of NNMT expression, cut-off value of the classification, and outcome endpoints. Three survival parameters (disease-free survival [DFS], progression-free survival [PFS], and relapse-free survival [RFS]) are adopted as the outcome endpoint in meta-analysis (Tian et al., 2017). Furthermore, we used time to tumor progression (TTP) to represent three survival parameters (DFS, PFS, and metastasis-free survival [MeFS]). Meanwhile, cancer-specific survival (CSS) and disease-specific survival (DSS) were combined and used as the same survival parameter. When studies only presented Kaplan-Meier curves to describe prognosis, we extracted statistical survival data using Engauge Digitizer V4.1, and calculated the HR and 95% CI using Tierney's method (Tierney et al., 2007).

### Assessment of study quality

We used the Newcastle-Ottawa Scale (NOS) (Wells et al., 2009) to assess study quality, which was previously adopted by Tian et al. and Zhou et al. (Tian et al., 2017; Zhou et al., 2018). Each study was judged on the basis of selection, comparability, and outcome assessment. The highest mark given was nine, and studies with an NOS score higher than six were viewed as high quality. Two authors (SL and LQ) separately evaluated the quality of these studies. Disagreements in study score were resolved through discussion. Where there was a disagreement on the assessment of study quality, the issue was resolved by discussion or consensus with the third investigator (ZY).

### Statistical analysis

In this study, HR and 95% CI were applied to evaluate the association between NNMT expression levels and the outcomes of patients with solid tumors. Statistical heterogeneity was assessed using *Q*-tests and the I-squared test, and *P* < 0.10 or *I*^2^ > 50% was considered as statistically significant heterogeneity. If there was heterogeneity among included studies, a random effect model was used to pool the HR; otherwise, a fixed effect model was utilized. Publication bias was assessed by Begg's funnel plot and Egger's regression method. Stata Version 12.0 (Stata Corporation, College Station, TX, USA) was used to analyze the data extracted from the studies. All statistical tests were two-sided, and *P* < 0.05 was defined as statistically significant.

## Results

### Search results and study characteristics

**Figure 1:**
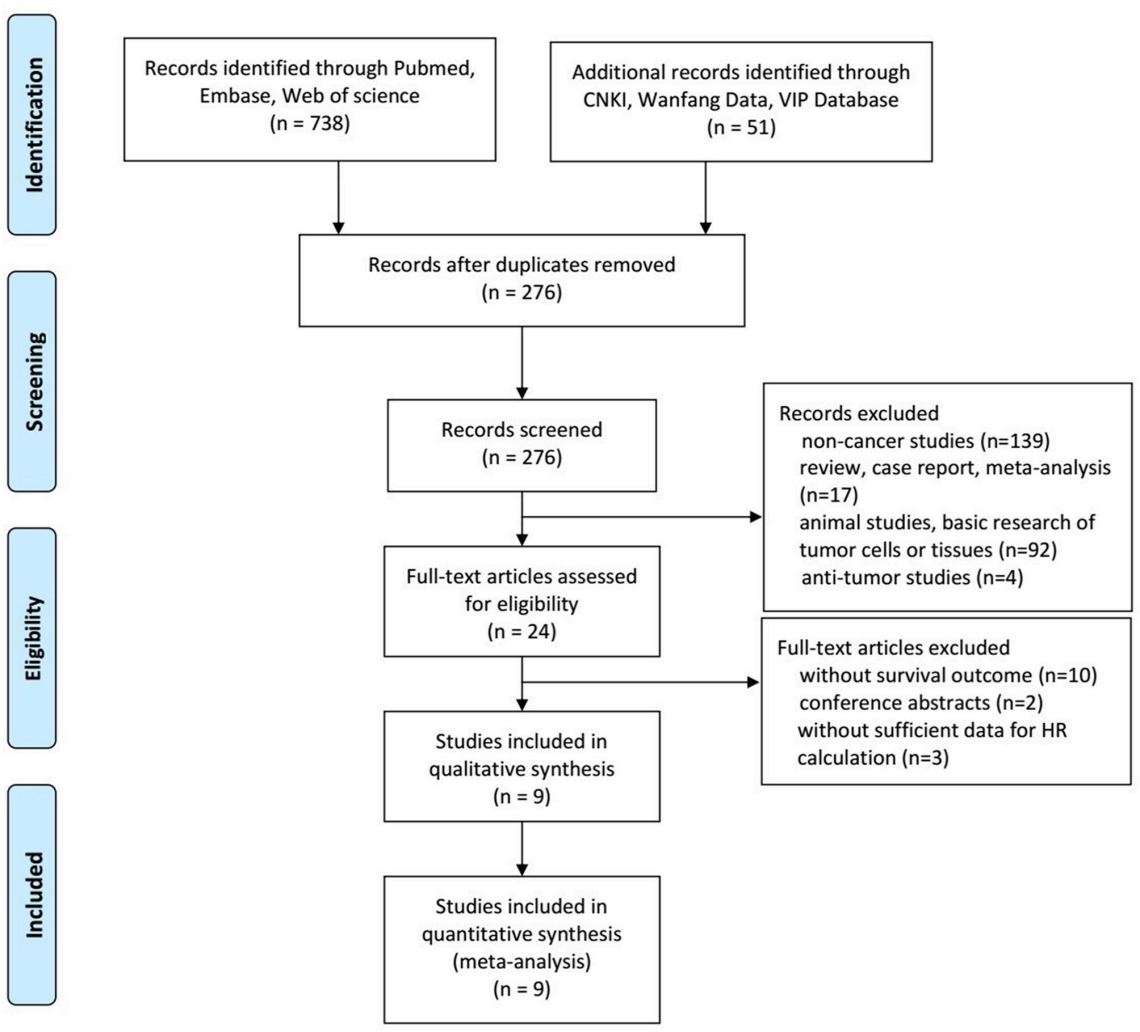
Flow diagram for selection of studies.

A total of 789 studies were initially identified by searching computerized databases. After screening titles and abstracts, we discarded 513 publications that were duplicated, and excluded 252 publications based on irrelevant subject matter. We reviewed 24 studies and found that 12 studies investigated the association between NNMT expression levels and prognosis in patients with solid tumors. However, three studies lacked sufficient data to calculate the HR and 95% CI. Finally, a total of 3,340 patients in nine studies including 14 cohorts were included (Figure 1).

The eligible studies were published from 2005 to 2017, and their clinical characteristics are summarized in Table 1. The patients enrolled in these studies were from China, Japan, Korea, and USA. In our study, we investigated the association between NNMT expression levels and prognosis for solid tumors (glioblastoma, gastric cancer, pancreatic cancer, prostate cancer, nasopharyngeal cancer, lung cancer, hepatocellular cancer, and renal cancer). The cut-off values are listed in Table 2.

**Table 1:** Characteristics of included studies.

**References**	**Type of cancer**	**Region**	**Case**	**NNMT (high/low)**	**Follow-up period Median(range)**	**Test method**	**Location**	**Outcome endpoints**	**NOS**
Jung et al., 2017 TCGA GBM	Glioblastoma	TCGA	525	264/261	NR	Microarray Platform HG-UG133A	Tumor	OS	6
Jung et al., 2017 TCGA GBMLGG	Glioblastoma	TCGA	667	334/333	NR	RNA-seq	Tumor	OS	6
Jung et al., 2017 Rembrandt	Glioblastoma	TCGA	397	194/203	NR	Microarray	Tumor	OS	6
Jung et al., 2017 Phillips	Glioblastoma	TCGA	77	38/39	NR	Microarray	Tumor	OS	6
Jung et al., 2017 Gravendeel	Glioblastoma	TCGA	270	133/137	NR	Microarray	Tumor	OS	6
Jung et al., 2017 Nutt	Glioblastoma	TCGA	50	25/25	NR	Microarray	Tumor	OS	6
Chen et al., 2016	Gastric Cancer	China	617	341/276	NR	IHC	Tumor	OS	8
Xu et al., 2016	Pancreatic Cancer	China	178	99/79	NR	IHC	Tumor	OS	9
Zhou et al., 2014	Prostate Cancer	China	81	50/31	52(7–96)	IHC	Tumor	PFS, OS	8
Bi et al., 2014	Pancreatic Cancer	America	22	14/8	NR	qPCR	Tumor	OS	7
Win et al., 2013	Nasopharyngeal Cancer	Taiwan China,	124	62/62	71(1–141)	IHC	Tumor	DSS, MeFS	8
Ujiie et al., 2012	Lung Cancer	Japan	109	27/82	NR	ELISA	Serum	OS	7
Kim et al., 2009	Hepatocellular Cancer	Korea	120	48/72	50(3–92)	qPCR	Tumor	OS, DFS	8
Yao et al., 2005	Renal Cancer	Japan	103	35/68	71.0(1.9–188.6)	qPCR	Tumor	CSS	9

*NR, not reported; IHC, immunohistochemistry; qPCR, quantitative polymerase chain reaction; ELISA, enzyme linked immunosorbent assay; TCGA, The Cancer Genome Atlas; NOS, Newcastle-Ottawa Scale; OS, overall survival; DSS, disease-specific survival; CSS, cancer-specific survival; PFS, progression-free survival; DFS, disease-free survival; MeFS, metastasis-free survival*.

**Table 2:** Cutoff value of high nicotinamide N-methyltransferase expression in eligible studies.

**References**	**Type of cancer**	**Test target**	**Cutoff for NNMT upregulation**
Jung et al., 2017 TCGA GBM	Glioblastoma	mRNA	High: Z-score > 0. Gene signature scores were calculated by deriving Z-scores across all the tumor specimens from single sample GSEA results for each gene set.
Jung et al., 2017 TCGA GBMLGG	Glioblastoma	mRNA	
Jung et al., 2017 Rembrandt	Glioblastoma	mRNA	
Jung et al., 2017 Phillips	Glioblastoma	mRNA	
Jung et al., 2017 Gravendeel	Glioblastoma	mRNA	
Jung et al., 2017 Nutt	Glioblastoma	mRNA	
Bi et al., 2014	Pancreatic Cancer	mRNA	High: mRNA levels > mean expression level.
Kim et al., 2009	Hepatocellular Cancer	mRNA	High: mRNA levels ≥ 4.40 (copy number ratio). Using the ΔCT value (NNMT CT - average CT of reference genes), the mRNA copy number ratio was calculated as 2-ΔCt.
Yao et al., 2005	Clear-cell Renal Cancer	mRNA	High: mRNA levels > mean expression level.
Chen et al., 2016	Gastric Cancer	Protein	High: H-score > 120. The staining intensity is scored according to 4 grades: 0 (no staining), 1 (weak staining), 2 (moderate staining), and 3 (intense staining). The product of the percentage of positive cells and the respective intensity scores are used as the final staining score.
Xu et al., 2016	Pancreatic Cancer	Protein	High: H-score ≥ 110. The staining intensity was scored as 0 (no staining), 1+(weak staining), 2+ (moderate staining), or 3+ (intense staining). Then the percentage of cells stained at the respective intensity was determined and multiplied by the intensity score to yield an intensity percentage score.
Zhou et al., 2014	Prostate Cancer	Protein	Immunoreactivity (IR) score > 4. The percentage scoring of immunoreactive tumor cells is as follows: 0, 0%; 1, <1%; 2, 1–10%; 3, 11–33%; 4, 34–67%; and 5, >67%. The staining intensity was visually scored and stratified as follows:0, none; 1, weak; 2, moderate; and 3, strong. The final NNMT immunohistochemical score is presented as a composite (intensity + extent).
Win et al., 2013	Nasopharyngeal Cancer	Protein	High: H score > median. H score is calculated using the following equation: H score = ∑Pi (*i* + 1), where *i* is the intensity (ranging from 0 to 4), and Pi is the percentage of stained tumor cells of intensity, varying from 0 to 100 %.
Ujiie et al., 2012	Lung Cancer	Protein	High: > median NNMT value (710 pg/ml).

*GSEA, Gene Set Enrichment Analysis*.

### Qualitative assessment

The Newcastle-Ottawa quality assessment scale was used to assess study quality. The included studies with scores ranging from 6 to 9 indicated that they had high methodological quality (Table 1).

### Association between NNMT expression levels and patient prognosis

**Figure 2:**
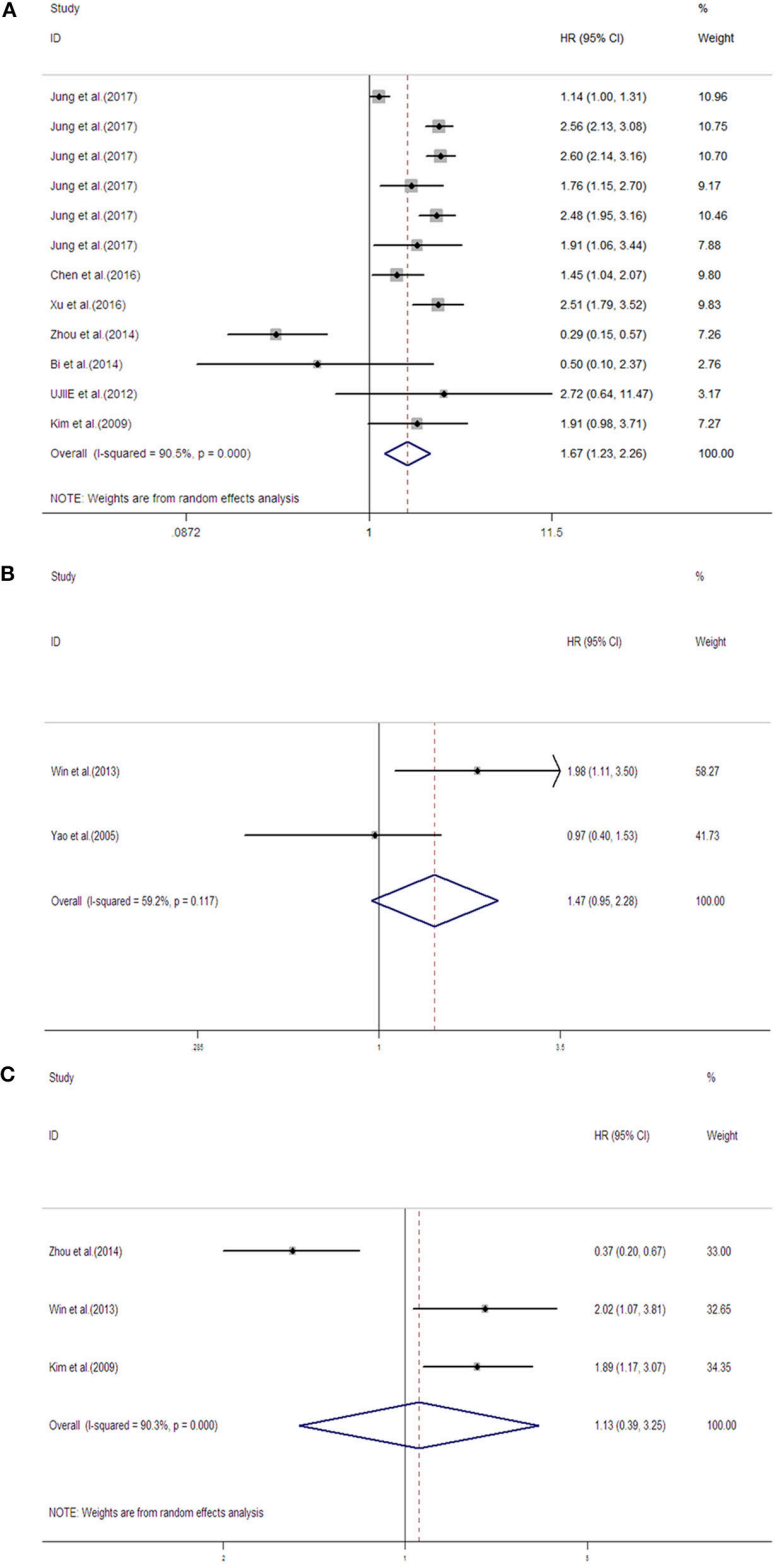
Meta-analysis (forest plot) of NNMT evaluation studies included in **(A)** OS, **(B)** DSS, and **(C)** TTP.

The seven studies (Kim et al., 2009; Ujiie et al., 2012; Bi et al., 2014; Zhou et al., 2014; Chen et al., 2016; Xu et al., 2016; Jung et al., 2017) assessed the association between NNMT expression levels and OS, while the two studies (Yao et al., 2005; Win et al., 2013) used DSS as an endpoint, and the three studies (Kim et al., 2009; Win et al., 2013; Zhou et al., 2014) used TTP as an endpoint. The estimated pooled HR for OS identified high NNMT expression levels as a predictor of poor prognosis in patients with solid tumors (HR _*high*/*low*_ = 1.67, 95% CI = 1.23–2.26). However, there was no significant association between NNMT expression levels and either DSS (HR = 1.47, 95% CI = 0.95–2.28) or TTP (HR = 1.13, 95% CI = 0.39–3.25). There was no significant heterogeneity in patients for whom DSS was measured (*I*^2^ = 59.2%, *P* = 0.12). However, heterogeneity may exist in patients where OS (*I*^2^ = 90.5%, *P* < 0.001) or TTP (*I*^2^ = 90.3%, *P* < 0.001) was measured (Figure 2).

### Subgroup analysis

As shown in Table 3, we conducted subgroup analyses (presence of glioblastoma, ethnicity, methods used to extract HR, test targets, and sample size) to explore the source of heterogeneity in our study.

**Table 3:** Subgroup analyses of the association between nicotinamide N-methyltransferase upregulation and overall survival for patients with solid tumor.

**Stratified analysis**	**No. of cohorts**	**Pooled HR (95% CI)**		***I*^2^ (%)**
		**Random effects**		
**CANCER TYPE**				
glioblastoma	6	2.00	(1.38–2.90)	93.6
**ETHNICITY**				
Asian	5	1.36	(0.68–2.72)	87.9
Non-Asian	7	1.89	(1.31–2.73)	92.6
**METHODS EXTRACTED HR**				
HR and 95% CI	4	1.23	(0.58–2.63)	90.8
Kaplan-Meier curves	8	1.92	(1.35–2.74)	91.4
**TEST TARGETS**				
mRNA-testing	8	1.89	(1.35–2.67)	91.4
protein-testing	4	1.25	(0.53–2.94)	90.8
**SAMPLE SIZE**				
≥200	5	1.94	(1.30–2.90)	95
<200	7	1.37	(0.77–2.45)	82.9

**Figure 3:**
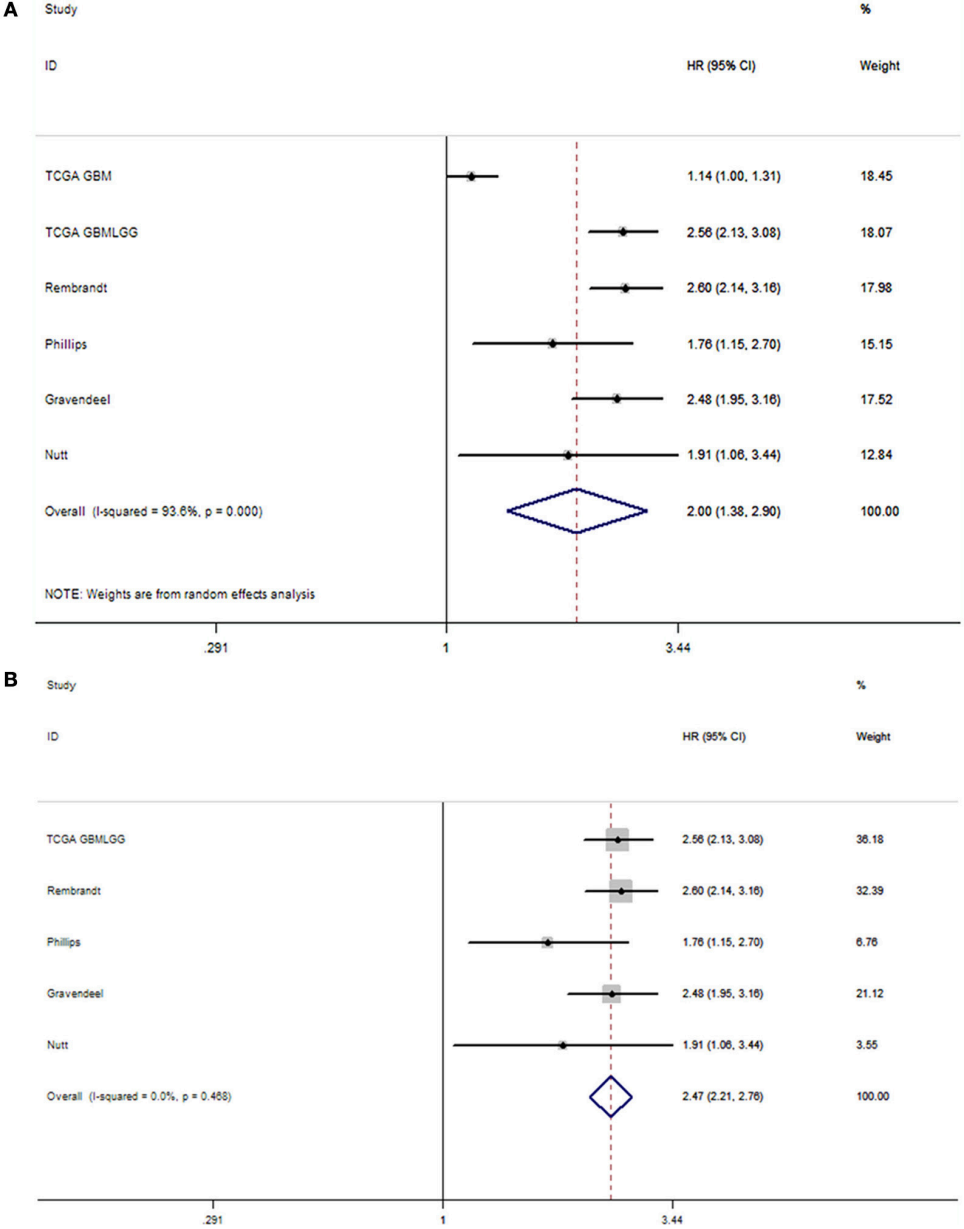
Forrest plot of the subgroup analysis for the effect of NNMT upregulation on OS in **(A)** patients with glioblastoma, **(B)** patients with glioblastoma (excluding TCGA GBM dataset).

Based on previous results obtained by Jung et al. (2017), we conducted a subgroup analysis focusing on the prognosis of patients with glioblastoma. The pooled HR showed that elevated NNMT levels were associated with poor prognosis (HR = 2.00, 95% CI = 1.38–2.90). Here, we also found significant heterogeneity in the patient population (*I*^2^ = 93.6%, *P* < 0.001). However, we re-evaluated the prognostic role of NNMT levels after excluding the outlier cohort (TCGA GBM dataset). Here, the pooled HR was 2.47 (95% CI = 2.21–2.76), and there was no more heterogeneity among the studies (*I*^2^ = 0%, *P* = 0.47) (Figure 3).

For our subgroup analysis of patient ethnicity, we classified studies reporting on OS into Asian and non-Asian groups. High NNMT expression levels were associated with unfavorable outcomes in non-Asian patients (HR = 1.89, 95% CI = 1.31–2.73); however, NNMT expression levels were not associated with OS in Asian patients (HR = 1.36, 95% CI = 0.68–2.72). Heterogeneity existed in both the Asian subgroup (*I*^2^ = 87.9%, *P* < 0.001) and in the non-Asian subgroup (*I*^2^ = 92.6%, *P* < 0.001) (Supplemental Figure 1).

For our subgroup analysis of HR extraction method, we first investigated survival statistic data calculated from Kaplan–Meier curves, and found that pooled HR was 1.92 (95% CI = 1.35–2.74), with significant heterogeneity (*I*^2^ = 91.4%, *P* < 0.001). Next, we investigated the HRs and 95% CIs directly from the included studies, and found that the pooled HR was 1.23 (95% CI = 0.58–2.63). This analysis also showed a significant amount of heterogeneity (*I*^2^ = 90.8%, *P* < 0.001) (Supplemental Figure 2).

Regarding the subgroup analysis of test targets, the studies were classified into either the mRNA-testing or protein-testing group. High NNMT expression levels were associated with unfavorable outcomes in the mRNA-testing subgroup (HR = 1.89, 95% CI = 1.35–2.67); however, NNMT expression levels were not associated with OS in the protein-testing subgroup (HR = 1.25, 95% CI = 0.53–2.94). Heterogeneity still existed in both the mRNA-testing (*I*^2^ = 91.4%, *P* < 0.001) and protein-testing groups (*I*^2^ = 90.8%, *P* < 0.001) (Supplemental Figure 3A). We re-evaluated the prognostic role of NNMT levels after excluding an outlier cohort (TCGA GBM dataset) in the mRNA-testing group, thus yielding a pooled HR of 2.43 (95% CI = 2.18–2.71). Here, we did not observe significant heterogeneity (*I*^2^ = 24.7%, *P* = 0.240) (Supplemental Figure 3B).

We also analyzed a subgroup based on sample size. Here, included studies were divided into two groups according to sample size: one group with a sample size of ≥200 patients and one group with a sample size of <200 patients (Tian et al., 2017). In the subgroup with a sample size of ≥200 patients, pooled HR was 1.94 (95% CI = 1.30–2.90) and heterogeneity existed (*I*^2^ = 95.0%, *P* < 0.001). In the subgroup with a sample size of <200, pooled HR was 1.37 (95% CI = 0.77–2.45), with significant heterogeneity (*I*^2^ = 82.9%, *P* < 0.001) (Supplemental Figure 4).

### Sensitivity analysis

**Figure 4:**
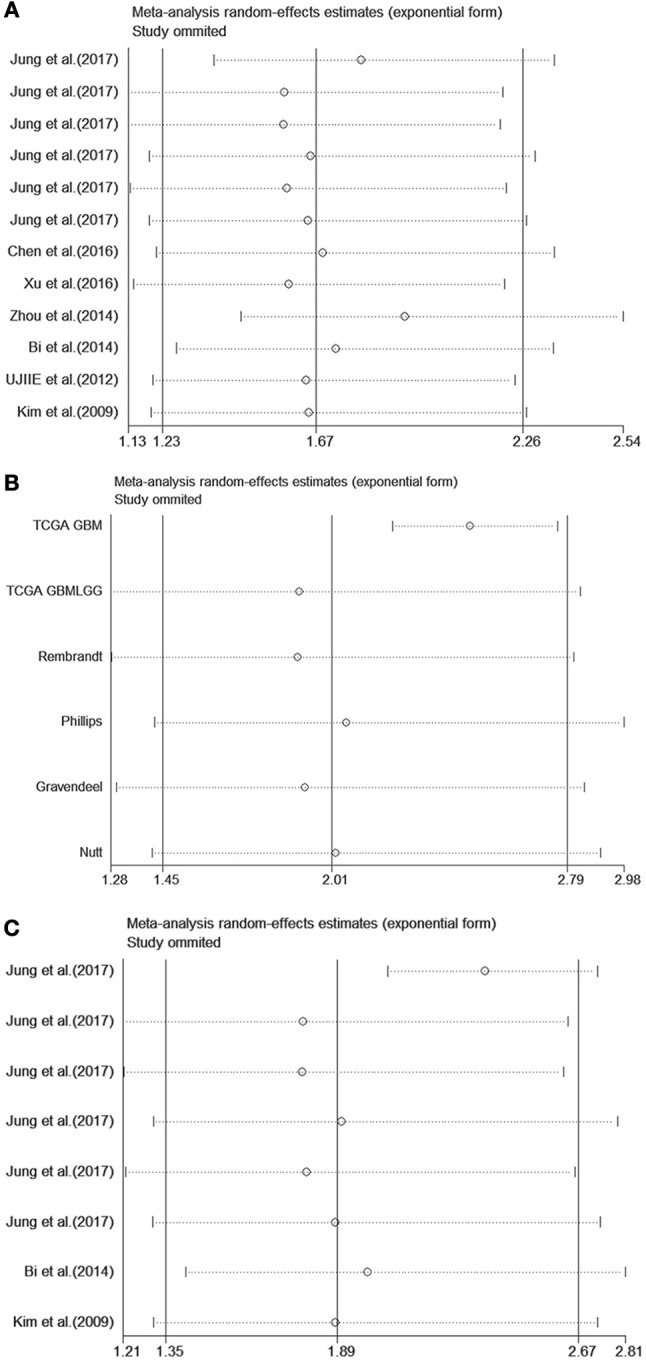
Sensitivity analysis for NNMT upregulation on OS in **(A)** patients with solid tumor, **(B)** subgroup of glioblastoma, **(C)** mRNA-testing group.

To perform sensitivity analysis, we eliminated one study at a time to test the robustness of our results. We conducted sensitivity analysis for OS in patients with solid tumors, the subgroup of patients with glioblastoma, and the mRNA-testing group. The pooled HRs were not significantly altered by excluding any single study that measured OS as an endpoint. However, the heterogeneity of pooled HR for OS was substantially reduced after removing the study conducted by Zhou et al. (2014) and the TCGA GBM dataset from Jung et al. (2017) (*I*^2^ = 44.5%, *P* = 0.062), indicating that these outlier cohorts may be the source of statistical heterogeneity. The combined HR for the remaining studies was 2.33 (95% CI = 2.11–2.58) (Supplemental Figure 5). Furthermore, the HR calculated from the TCGA GBM dataset affected the results in the subgroup of patients with glioblastoma, as well as the mRNA-testing group (Figure 4).

### Publication bias

**Figure 5:**
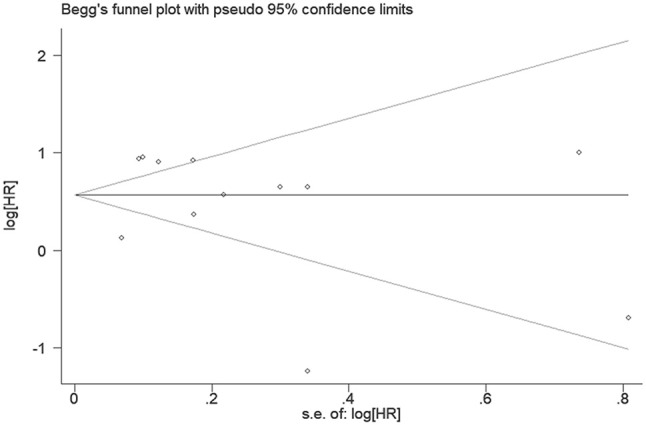
Funnel plots of included studies in the analysis of NNMT expression and the prognosis for solid tumors.

Begg's funnel plot combined with Egger's test was applied to assess the publication bias for OS in patients with solid tumors. Our results revealed no evidence of publication bias (Begg's test, *P* = 0.15; Egger's test, *P* = 0.90) (Figure 5).

## Discussion

NNMT is strongly expressed in liver, and expressed in adipose tissue, kidney, lung, and skeletal muscle in low levels (Sugawara et al., 2005). Variations in NNMT expression correlate with patient prognosis in cancer. Here, we confirmed that high NNMT expression levels correlate with poor prognosis in patients with solid tumors. To the best of our knowledge, this is the first study to investigate the prognostic value of NNMT expression in patients with solid tumors.

The association between NNMT expression levels and prognosis in patients with solid tumors has been previously investigated; however, data regarding the prognostic value of NNMT were conflicting. Xu et al. found that, in patients with pancreatic cancer, high NNMT expression levels are associated with shorter OS, and correlate with unfavorable clinicopathological features (Xu et al., 2016). Similarly, NNMT upregulation has been shown to enhance cell migration and invasion (Yu et al., 2015). Bi et al. also reported that NNMT mRNA levels are significantly elevated in pancreatic cancer cells, but, in their study, NNMT expression levels do not correlate with OS (Bi et al., 2014). In renal carcinoma, NNMT mediates cell invasion and metastasis by activating the PI3K/Akt/SP1/MMP-2 pathway (Tang et al., 2011); however, Yao et al. found that survival rates do not correlate with NNMT expression levels (Yao et al., 2005). Additionally, in patients with glioblastoma, Jung et al. showed that high NNMT expression levels are not associated with prognosis in TCGA GBM dataset, however, NNMT upregulation is found to be correlated with poor prognosis in five other cohorts, including Rembrandt, TCGA GBMLGG, Phillips, and Gravendeel, and Nutt (Jung et al., 2017). Win et al. reported that NNMT upregulation predicts poor patient prognosis in nasopharyngeal cancer, and that NNMT overexpression is associated with Akt phosphorylation level (Win et al., 2013). Chen et al. showed that high NNMT expression is an independent prognostic factor associated with poor OS in gastric carcinoma (Chen et al., 2016). In lung cancer, Ujiie et al. showed that NNMT levels are not associated with OS (Ujiie et al., 2012); however, Zhou et al. showed that high NNMT levels are associated with prolonged PFS and OS in prostate cancer (Zhou et al., 2014). Finally, Kim et al. found that NNMT expression levels correlate with tumor stage in hepatocellular carcinoma. Patients with higher levels of NNMT tend to have shorter survival time, although the difference is not statistically significant. However, in this study, higher NNMT expression levels are significantly associated with shorter DFS (Kim et al., 2009).

To reduce heterogeneity in our study, we conducted subgroup and sensitivity analyses to explore the source of the observed heterogeneity. Firstly, we conducted a subgroup analysis of patient ethnicity, and classified the studies into either Asian or non-Asian groups. Different heritage backgrounds and dietary habits between Asian and Western populations contribute to different cancer histopathology and survival outcomes. Squamous cell cancer is the most prominent type of esophageal cancer in Asia; however, esophageal adenocarcinoma predominately affects Caucasian men (Zhang et al., 2012). Fukagai et al. showed that Japanese patients who have received hormonal treatment have long overall survival compared with Caucasian patients. Race also acts as an important prognostic factor in patients with prostate cancer (Fukagai et al., 2006) and pancreatic cancer. The median survival time after diagnosis for patients with pancreatic cancer is longer in Asian patients than that in Caucasians; whereas, the proportion of patients with papillary carcinomas or mucinous cystadenocarcinomas is higher in Asians than that in Caucasians or blacks (Longnecker et al., 2000). Finally, a study by Jung et al. investigated prognosis for patients with glioblastoma in six major databases (Jung et al., 2017); thus, we chose to conduct a subgroup analysis.

In our sensitivity analysis, we found that the study conducted by Zhou et al. (2014) and the TCGA GBM dataset from Jung et al. (2017) may be the source of heterogeneity. Zhou et al. found significantly elevated levels of NNMT in high-grade prostatic intraepithelial neoplasia and well-differentiated prostate cancer compared with benign prostate hyperplasia, indicating that NNMT may play different roles during tumorigenesis and tumor progression in prostate cancer. However, this study only focused on 81 patients with advanced prostate cancer. Moreover, abnormal testosterone levels are implicated in the progression of prostate cancer. NNMT can regulate sex-dependent methylation in mouse, implying NNMT expression may be influenced by alterations in sex hormone levels (Takasugi et al., 2013). Notably, the effects of sex hormones in prostate cancer differ from their effects in other types of solid tumors. With regard to the TCGA GBM dataset from Jung et al. (2017), the data taken from The Cancer Genome Atlas (TCGA) lacks sufficient clinicopathological information about the patients. Thus, heterogeneity may be caused by biological types of solid tumors, as well as by the clinicopathological staging of patients.

There were some limitations to our study. Firstly, different experimental methods were used to detect NNMT expression levels, which may cause inter-study heterogeneity. Secondly, we searched references published in English and Chinese, which may have led to publication bias. Thirdly, the survival data of six cohorts were obtained from online databases. Finally, because some studies did not provide HR and 95% CI directly, we calculated HR from the obtained survival curves.

## Conclusions

In summary, our meta-analysis indicated that high NNMT expression levels may be used as a prognostic predictor of OS in patients with solid tumors. More studies are still needed to evaluate the prognostic role of NNMT expression for each type of solid tumor.

## Author contributions

SL collected and analyzed the data, and wrote the paper. LQ analyzed the data, and participated in the writing of manuscript. ZY assisted with the data analyses. CH conceived and designed this study. All authors reviewed the paper, read and approved the final manuscript.

### Conflict of interest statement

The authors declare that the research was conducted in the absence of any commercial or financial relationships that could be construed as a potential conflict of interest.

## Supplementary material

Supplemental Figure 1 Forrest plot of the subgroup analysis (ethnicity) for the effect of NNMT upregulation on OS in patients with solid tumor.

Supplemental Figure 2 Forrest plot of the subgroup analysis (methods extracted HR) for the effect of NNMT upregulation on OS in patients with solid tumor.

Supplemental Figure 3 Forrest plot of the subgroup analysis (test targets) for the effect of NNMT upregulation on OS in **(A)** patients with solid tumor, **(B)** patients with solid tumor (excluding TCGA GBM dataset).

Supplemental Figure 4 Forrest plot of the subgroup analysis (sample size) for the effect of NNMT upregulation on OS in patients with solid tumor.

Supplemental Figure 5 Meta-analysis of the correlation between NNMT and OS across homogenous studies excluding Zhou's study and TCGA GBM dataset.
