# Supplementary figures and images for: Prognostic Value of Nicotinamide N-Methyltransferase Expression in Patients With Solid Tumors: A Systematic Review and Meta-Analysis
Source: Front Physiol. 2018 Oct 8;9:1407. doi: 10.3389/fphys.2018.01407 (PMC6187113; doi:10.3389/fphys.2018.01407)

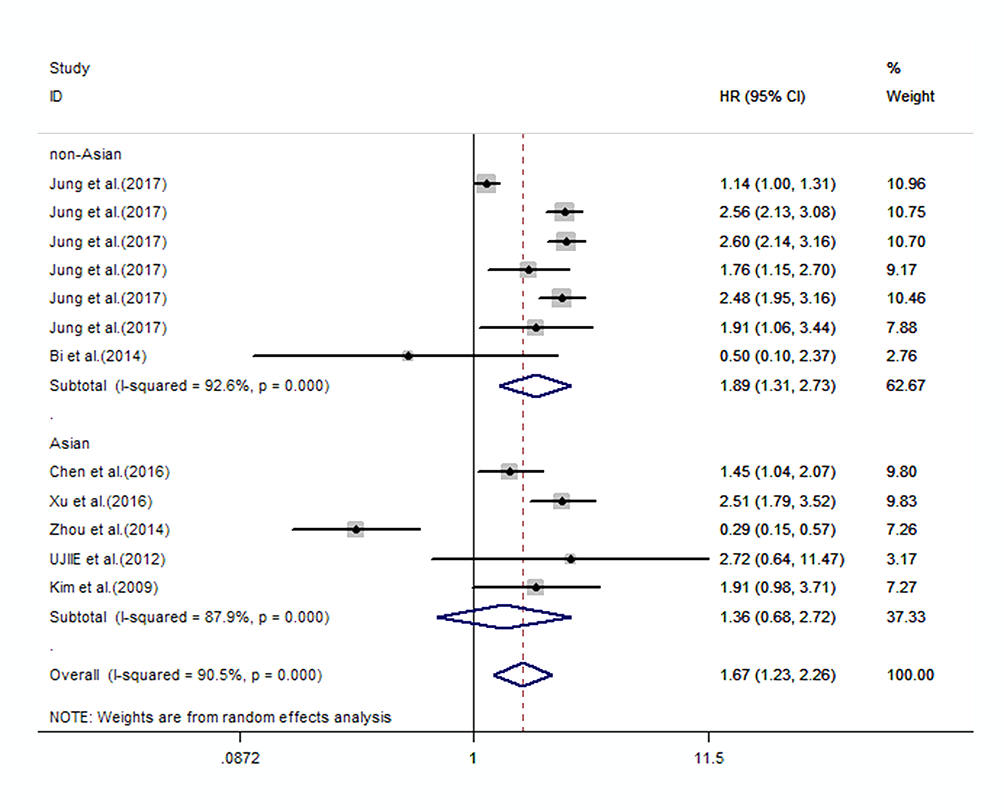

Supplement: Supplemental Figure 1 — Forrest plot of the subgroup analysis (ethnicity) for the effect of NNMT upregulation on OS in patients with solid tumor. [file Image_1.TIF]

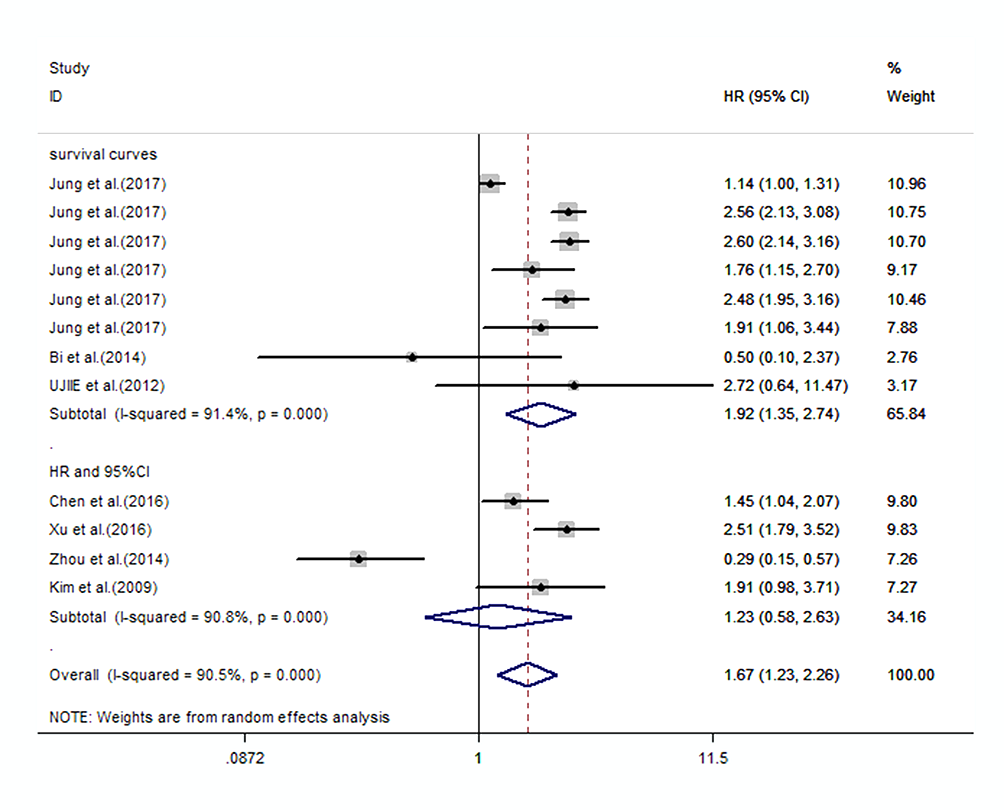

Supplement: Supplemental Figure 2 — Forrest plot of the subgroup analysis (methods extracted HR) for the effect of NNMT upregulation on OS in patients with solid tumor. [file Image_2.TIF]

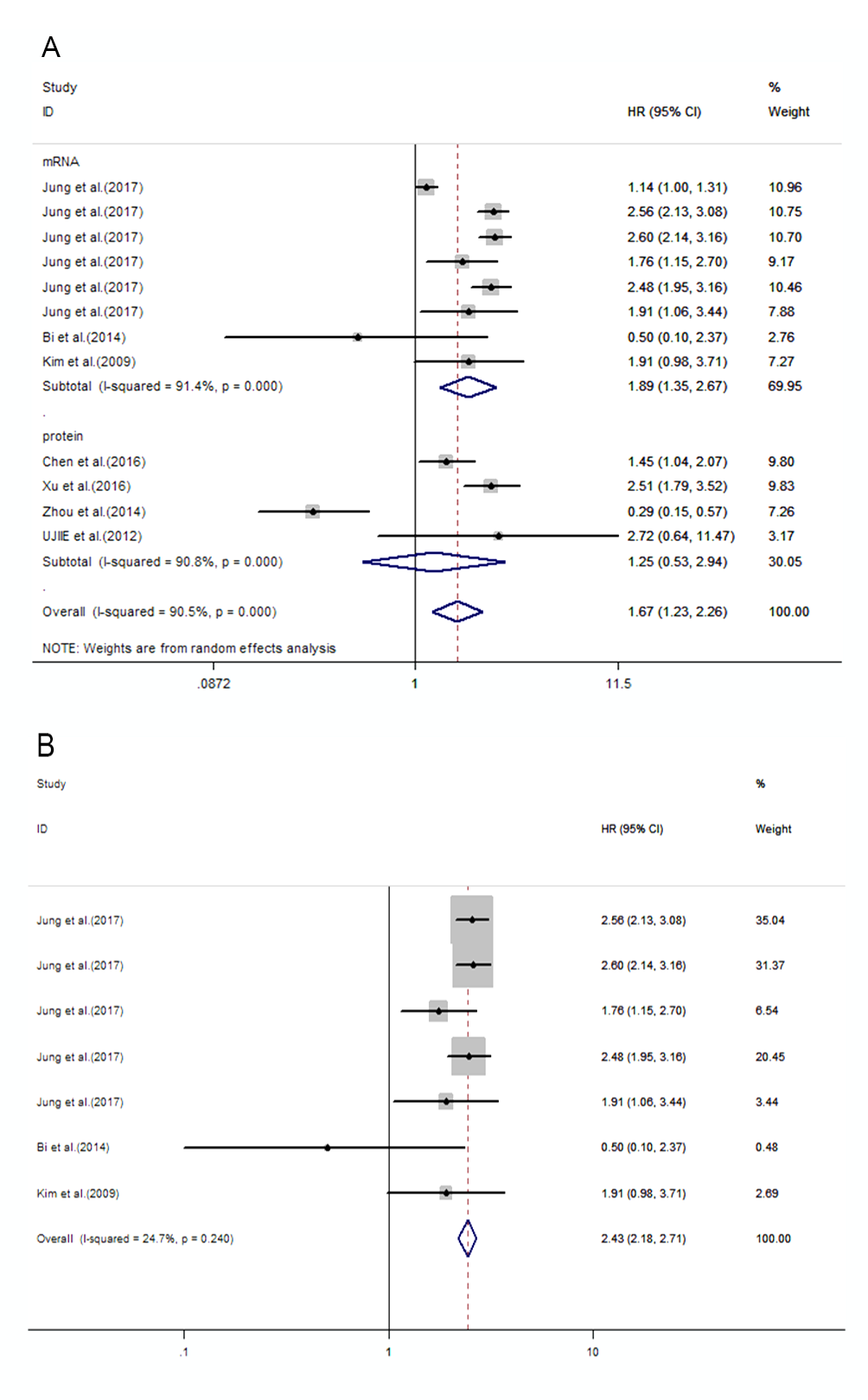

Supplement: Supplemental Figure 3 — Forrest plot of the subgroup analysis (test targets) for the effect of NNMT upregulation on OS in (A) patients with solid tumor, (B) patients with solid tumor (excluding TCGA GBM dataset). [file Image_3.tif]

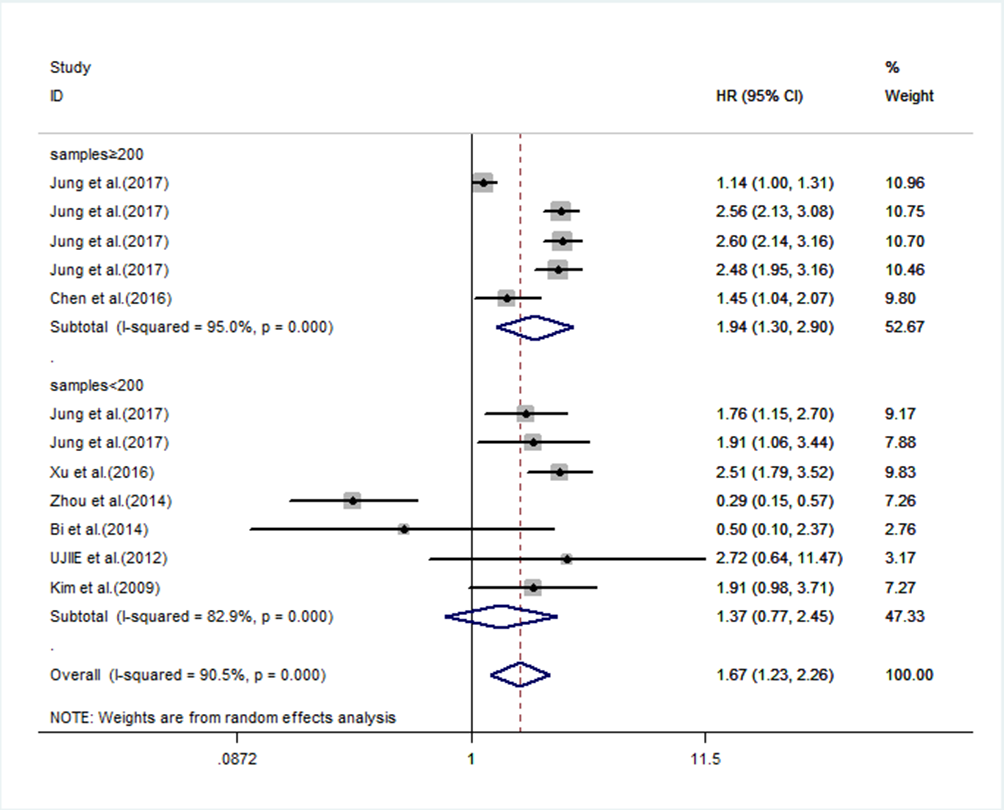

Supplement: Supplemental Figure 4 — Forrest plot of the subgroup analysis (sample size) for the effect of NNMT upregulation on OS in patients with solid tumor. [file Image_4.tif]

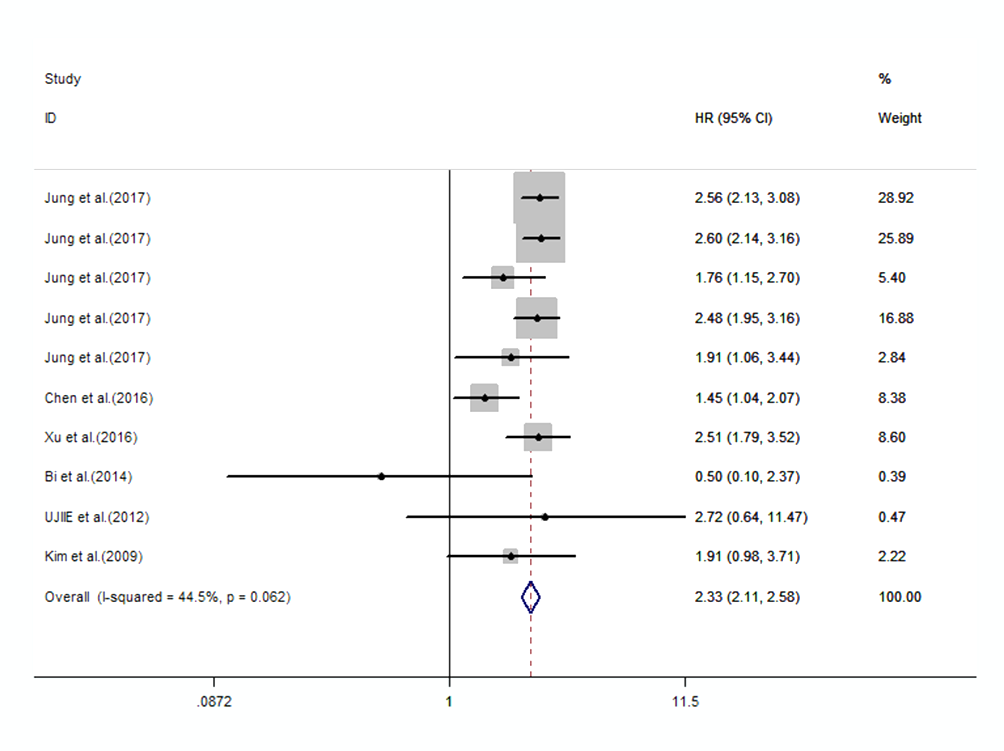

Supplement: Supplemental Figure 5 — Meta-analysis of the correlation between NNMT and OS across homogenous studies excluding Zhou's study and TCGA GBM dataset. [file Image_5.tif]
